# Impact of clinical targeted sequencing on endocrine responsiveness in estrogen receptor-positive, HER2-negative metastatic breast cancer

**DOI:** 10.1038/s41598-021-87645-6

**Published:** 2021-04-14

**Authors:** Kanako Hagio, Toraji Amano, Hideyuki Hayashi, Takashi Takeshita, Tomohiro Oshino, Junko Kikuchi, Yoshihito Ohhara, Ichiro Yabe, Ichiro Kinoshita, Hiroshi Nishihara, Hiroko Yamashita

**Affiliations:** 1 Department of Breast Surgery, Hokkaido University Hospital, Kita 14, Nishi 5, Kita-ku, Sapporo, 060-8648 Japan; 2 Clinical Research and Medical Innovation Center, Hokkaido University Hospital, Sapporo, Japan; 3 Division of Clinical Cancer Genomics, Hokkaido University Hospital, Sapporo, Japan; 4 Genomics Unit, Keio Cancer Center, Keio University School of Medicine, 35 Shinanomachi, Shinjukuku, Tokyo, 160-8582 Japan; 5 Division of Clinical Genetics, Hokkaido University Hospital, Sapporo, Japan

**Keywords:** Breast cancer, Cancer genomics

## Abstract

Clinical targeted sequencing allows for the selection of patients expected to have a better treatment response, and reveals mechanisms of resistance to molecular targeted therapies based on actionable gene mutations. We underwent comprehensive genomic testing with either our original in-house CLHURC system or with OncoPrime. Samples from 24 patients with estrogen receptor-positive, human epidermal growth factor receptor 2-negative metastatic breast cancer underwent targeted sequencing between 2016 and 2018. Germline and somatic gene alterations and patients’ prognosis were retrospectively analyzed according to the response to endocrine therapy. All of the patients had one or more germline and/or somatic gene alterations. Four patients with primary or secondary endocrine-resistant breast cancer harbored germline pathogenic variants of *BRCA1*, *BRCA2*, or *PTEN*. Among somatic gene alterations, *TP53*, *PIK3CA*, *AKT1*, *ESR1*, and *MYC* were the most frequently mutated genes. *TP53* gene mutation was more frequently observed in patients with primary endocrine resistance compared to those with secondary endocrine resistance or endocrine-responsive breast cancer. Recurrent breast cancer patients carrying *TP53*-mutant tumors had significantly worse overall survival compared to those with *TP53*-wild type tumors. Our 160-gene cancer panel will be useful to identify clinically actionable gene alterations in breast cancer in clinical practice.

## Introduction

Although recent advances in therapeutic strategies have improved survival in breast cancer, metastatic breast cancer is an incurable disease. Genomic alterations in tumors are acquired during the development from early to late stages^1–3^. While there are numerous reports of genomic features in early and metastatic breast cancers^4–7^, they have not conclusively demonstrated the clinical usefulness of genomic sequencing for treatment decisions. Clinical targeted sequencing facilitated by next-generation sequencing (NGS), identifies genetically-based drivers and pathways to help develop and apply valid molecular-targeted therapies. Furthermore, it might reveal mechanisms of resistance to molecular-targeted therapies^8^.

Breast cancer is the most common malignancy in women. Almost 80% of breast cancers are classified into the estrogen receptor (ER)-positive subtype^9^. Endocrine therapy for ER-positive breast cancer was one of the earliest targeted treatments. The clinical benefit rate for highly endocrine-responsive disease can be up to 70–80%^10^. Furthermore, endocrine therapy causes no severe side effects and maintains or improves quality of life. However, the resistance to endocrine therapy is an unavoidable problem especially in metastatic breast cancer. We previously reported that endocrine responsiveness after recurrence was a key issue for improved post-relapse survival in ER-positive recurrent breast cancer^11^. Recently, inhibitors of molecules within well-defined signaling pathways, including mammalian target of rapamycin (mTOR), cyclin-dependent kinase 4/6 (CDK4/6), phosphatidylinositol 3 kinase (PI3K), and akt murine thymoma viral oncogene (AKT), have been combined with endocrine agents to overcome resistance to endocrine therapy^12,13^. Understanding predictive factors for the response to endocrine therapy will facilitate the selection of patients to be treated with specific targeted therapies in ER-positive breast cancer. The 4th European School of Oncology (ESO)-European Society for Medical Oncology (ESMO) International Consensus Guidelines for Advanced Breast Cancer (ABC 4) defines endocrine resistance as primary and secondary endocrine resistances^14^. Since the molecular characteristics of tumors differ according to endocrine responsiveness^15^, the investigation of actionable gene alterations based on response to endocrine therapy will help to select appropriate treatments in ER-positive metastatic breast cancer.

In April 2016, an original clinical sequencing system named ‘CLHURC’ for patients with cancer was started at our hospital^16–20^. Targeted sequencing of 160 cancer-related genes using genomic DNA from both tumor tissue and blood was performed under the CLHURC framework. We previously reported the clinical utility of ‘CLHURC’ genomic testing for patients with meningioma^16^, thyroid carcinoma^17^, sinonasal papilloma^18^, pancreatic cancer^19^, and various other cancers such as colorectal, breast, stomach, and lung cancers^20^. The ‘CLHURC’ system has been proven to be safe and secure, and could be feasible and promising for application in clinical practice. Here, we report our experience of clinical targeted sequencing in ER-positive, human epidermal growth factor receptor 2 (HER2)-negative advanced breast cancer. We sought to verify the clinical utility of our 160-gene cancer panel for identifying clinically actionable gene alterations in patients with endocrine-responsive and endocrine-resistant breast cancer.

## Results

### Characteristics of patients

Characteristics of patients at the time of clinical sequencing are summarized in Table 1, and detailed information including disease-free and overall survivals according to endocrine responsiveness is shown in Table 2. Of the 24 patients with ER-positive, HER2-negative metastatic breast cancer, ten (42%) patients had a family history of breast and/or ovarian cancers. Eighteen (75%) patients had recurrent breast cancer and four (17%) patients presented with Stage IV disease. Tumor samples were obtained from the primary tumors of 13 (54%) patients and from the metastatic tumors of 11 (46%) patients. At the time of clinical sequencing, 15 (63%) patients were undergoing endocrine therapy; six (25%) as first-line, four (17%) as second-line, and five (21%) as third-line or later. Endocrine responsiveness in metastatic breast cancer was classified as primary endocrine resistance in seven (29%) patients, secondary endocrine resistance in eight (33%) patients, and endocrine-responsive breast cancer in nine (33%) patients.

**Table 1:** Characteristics of patients at the time of clinical sequencing.

Characteristics	Patients (n = 24)
Age, median (range), years	65.5 (33–73)
**Sex**	
Female	24 (100%)
Male	0
**ECOG performance status**	
0	13 (54%)
1	7 (29%)
2	2 (8%)
3	2 (8%)
**Family history**	
Breast cancer and/or ovarian cancer	10 (42%)
Other cancer	8 (33%)
No family history of cancer	6 (25%)
**Smoking history**	
Yes	8 (33%)
No	16 (67%)
**Initial stage of diagnosis**	
I	1 (4%)
II	11 (46%)
III	6 (25%)
IV	4 (17%)
Unknown	2 (8%)
**Histopathology**	
Invasive ductal carcinoma	23 (96%)
Invasive lobular carcinoma	1 (4%)
**Site of the sample used for clinical sequencing**	
Primary tumor	13 (54%)
Metastatic tumor	11 (46%)
**Systemic therapy for metastatic breast cancer**	
First-line endocrine therapy	6 (25%)
Second-line treatment	
Endocrine therapy	4 (17%)
Chemotherapy	2 (8%)
Third-line or later	
Endocrine therapy	5 (21%)
Chemotherapy	7 (29%)
**Endocrine responsiveness in metastatic breast cancer**	
Primary endocrine resistance	7 (29%)
Secondary endocrine resistance	8 (33%)
Endocrine-responsive breast cancer	9 (38%)
**Platform used for clinical sequencing**	
CLHURC (in-house)	22 (92%)
OncoPrime	2 (8%)

**Table 2:** Characteristics, treatment, and survivals in 24 patients according to endocrine responsiveness.

No	Age^a^	Family history	Initial Stage	ER	Adjuvant endocrine therapy	DFS	First site of metastatic disease	Age^b^	Site of sample	Platform	OS
**Primary endocrine resistance**											
1	42	Other	II	100	None	1	Lymph node, bone	44	Primary	CLHURC	33
2	33	Breast/ovary	III	5	None	2	Lymph node, lung, liver, spleen	33	Primary	OncoPrime	12
3	67	Other	II	80	AI	3	Chest wall	70	Primary	CLHURC	42
4	41	Breast/ovary	II	90	TAM, OFS	16	Lung	44	Primary	CLHURC	57
5	48	Other	−	−	TAM	7	Lymph node	67	Metastasis (ovary)	CLHURC	> 269
6	42	Breast/ovary	II	95	TAM	11	Bone	45	Metastasis (chest wall)	OncoPrime	46
7	61	None	II	100	AI	23	Liver, bone	66	Metastasis (liver)	CLHURC	71
**Secondary endocrine resistance**											
8	61	Other	II	> 65	AI	36	−	65	Primary	CLHURC	−
9	58	Breast/ovary	III	100	TAM	54	Chest wall	72	Primary	CLHURC	187
10	63	Breast/ovary	II	−	TAM → AI	60	Chest wall	68	Primary	CLHURC	78
11	32	Breast/ovary	IV	> 65	N/A	N/A	Bone	33	Primary	CLHURC	20
12	66	None	IV	100	N/A	N/A	Lymph node, pleura	70	Primary	CLHURC	71
13	43	Breast/ovary	III	100	TAM	56	Lymph node	50	Metastasis (lymph node)	CLHURC	107
14	51	Other	II	−	TAM → AI	58	Lymph node	64	Metastasis (lung)	CLHURC	> 188
15	28	Breast/ovary	II	> 65	TAM	65	Lymph node, bone	38	Metastasis (liver)	CLHURC	114
**Endocrine-responsive breast cancer**											
16	61	Breast/ovary	I	> 65	TAM	97	Lymph node, bone	70	Primary	CLHURC	> 159
17	46	Other	III	60	AI	128	−	57	Primary	CLHURC	−
18	39	None	IV	> 65	N/A	N/A	Bone	46	Primary	CLHURC	126
19	72	Other	IV	90	N/A	N/A	Lymph node, bone, pleura	72	Primary (ILC)	CLHURC	> 49
20	57	None	III	100	AI → TAM	74	−	66	Metastasis (bone)	CLHURC	−
21	58	None	III	−	AI	77	−	68	Metastasis (liver)	CLHURC	−
22	39	Breast/ovary	−	−	TAM	124	Lung	59	Metastasis (lung)	CLHURC	> 204
23	59	Other	II	100	TAM → AI	148	Brain	73	Metastasis (brain)	CLHURC	> 205
24	39	None	II	95	TAM	247	Lymph node	66	Metastasis (lymph node)	CLHURC	361

*ER* estrogen receptor (% of positive cells), *DFS* disease-free survival (months), *OS* overall survival (months), *AI* aromatase inhibitor, *TAM* tamoxifen, *OFS* ovarian function suppression, *ILC* invasive lobular carcinoma, *N/A* not applicable, − unknown.

^a^Age at the time of initial diagnosis (years).

^b^Age at the time of clinical sequencing (years).

### Germline and somatic gene alterations

Detailed information regarding germline and somatic gene alterations according to endocrine responsiveness is shown in Table 3. All of the patients had one or more germline and/or somatic gene alterations. Germline pathogenic variants were identified in four (17%) patients: *BRCA1* in one patient (No. 13), *BRCA2* in two patients (No. 4 and No. 15), and *PTEN* in one patient (No. 11). All four patients harboring germline pathogenic variants had family history of breast and/or ovarian cancers. Somatic gene alterations were not detected in one patient (No. 11) who instead has a germline *PTEN* pathogenic variant. Germline pathogenic variants were not detected in patients with endocrine-responsive breast cancer.

**Table 3:** Germline and somatic gene alterations in 24 patients according to endocrine responsiveness.

No	Age	Germline pathogenic variant	Somatic gene alteration		
			Actionable gene mutation	Copy-number variant	
				Gain	Loss
**Primary endocrine resistance**					
1	44		*TP53* T253Nfs*11, *PIK3CA* V105_R108del		
2	33		*TP53* Y220C, *BRCA1* L63*		
3	70		*TP53* A161D, *TERT* V299M	*ESR1, MYC, CDK4, ECT2L, TNFAIP3*	
4	44	*BRCA2* T630Nfs*6	*DICER1* A518V		
5	67		*PIK3CA* H1047L, *TSC1* A84T	*FAM46C*, *NRAS*	
6	45		*TP53* E11Q, *STK11* T189l, *CEBPA* S190P		
7	66		*TP53* E339*, *PIK3CA* H1047R, *ESR1* D538G, MAP2K4 R75Tfs*7		
**Secondary endocrine resistance**					
8	65		*AKT1* E17K, *ESR1* D538G		
9	72		*TP53* Q192*, *PIK3CA* C420R, *ESR1* G415_C417del, *BRAF* R389C, *ATM* S1863F	*MYC, GNAS*	
10	68				*CRLF2, SMO*
11	33	*PTEN* R130*			
12	70		*RB1* R621S		
13	50	*BRCA1* Q1447Rfs*22	*AKT1* E17K, *CREBBP* Q1259*, *NF1* W561*		*CDKN2A*
14	64		*ESR1* G442R		
15	38	*BRCA2* D252Vfs*24	*ERBB2* G727A	*MYC, TERT*	*BRCA2*, *ARID1A, RB1*
**Endocrine-responsive breast cancer**					
16	70		*PIK3CA* H1047R, *CSF1R* A245T, *ARID1A* Q1334del, *FANCA* P1411L, *SF3B1* K700E		
17	57		*PIK3R1* R534Q, *AKT1* E17K, *PTEN* L325F		
18	46		*PIK3CA* E453K, *KIT* V540L		
19	72		*PIK3CA* H1047R		
20	66		*AKT1* E17K, *ROS1* D2213E, *FLT3* S454L, *DICER1* S678F, *MAP3K1 N1305D*, MAP3K1 N749Ifs*12		
21	68		*ESR1* E380Q, *HIST1H3B* D82Y	*PRKAR1A, BRIP1, CD79B*	
22	59		*AKT1* E17K		
23	73		*TP53* E11Q, *ASXL1* E1033V	*HIST1H3B, PRKAR1A, DAXX*	
24	66		*AKT1* E17K, *DDR2* T283I	*KLF6*	

*Age* age at the time of clinical sequencing (years).

Somatic gene alterations were identified in 23 (96%) of tumors (Table 3): *TP53* (7 tumors, 29%), *PIK3CA* (7 tumors, 29%), *AKT1* (6 tumors, 25%), and *ESR1* (6 tumors, 25%) were the most frequently mutated genes. The median numbers of germline and/or somatic gene alterations were three (range 2–7) in primary endocrine resistance, two (range 1–7) in secondary endocrine resistance, and three (range 1–6) in endocrine-responsive breast cancer. *RB1* gene alterations were detected in two tumors with secondary endocrine resistance (*RB1* R621S in the primary tumor of No. 12 and *RB1* loss in the metastatic tumor of No. 15). A patient (No. 2) whose primary tumor carried a *TP53* missense mutation (*TP53* Y220C) and a *BRCA1* nonsense mutation (*BRCA1* L63*) did not harbor germline *BRCA1* pathogenic variants.

Among somatic gene alterations, missense mutation was the most frequently detected mutation type. Mutation types found within the *TP53* gene were missense in four tumors (Y220C in No. 2, A161D in No. 3, and E11Q in No. 6 and No. 23), nonsense in two tumors (E339* in No. 7, and Q192* in No. 9), and frameshift in one tumor (T253Nfs*11 in No. 1). Mutation types found within the *PIK3CA* gene were missense in six tumors (H1047L in No. 5, H1047R in No. 7, 16, and 19, C420R in No. 9, E453K in No. 18) and in-frame deletion in one tumor (V105_R108del in No. 1). All six tumors with *AKT1* gene alterations had the E17K missense mutation. Mutation types found within the *ESR1* gene were missense in four tumors (D538G in No. 7 and No. 8, G442R in No. 14, and E380Q in No. 21), in-frame deletion in one tumor (G415_C417del in No. 9), and amplification in one tumor (No. 3).

Copy number variations such as copy number gain and copy number loss were identified as somatic mutations in nine tumors. Copy number gain was observed in seven (29%) tumors. All *MYC* gene alterations in three tumors corresponded to copy number gain (No. 3, 9, and 15). Copy number loss was identified in three (13%) tumors (No. 10, 13, and 15). A patient (No. 10) whose tumor had copy number loss of the *CRLE2* and *SMO* genes did not carry other somatic gene mutations nor germline pathogenic variants.

*TP53* gene mutation either in a primary or metastatic site was more frequently observed in patients with primary endocrine resistance compared to those with secondary endocrine resistance or endocrine-responsive breast cancer (*P* = 0.014, Table 4). In contrast, the frequencies of alterations in the *PIK3CA*, *AKT1*, *ESR1*, and *MYC* genes were not correlated with a particular endocrine-responsive status (Table 4). In addition, the frequencies of alterations in the *TP53*, *PIK3CA*, *AKT1*, *ESR1*, and *MYC* genes were similar in samples taken from primary or metastatic sites (Table 5).

**Table 4:** Number of patients harboring somatic gene alterations according to endocrine responsiveness.

	Primary resistance (n = 7)	Secondary resistance (n = 8)	Responsive (n = 9)	*P* value
*TP53*	5 (71%)	1 (13%)	1 (11%)	0.014*
*PIK3CA*	3 (43%)	1 (13%)	3 (33%)	0.41
*AKT1*	0	2 (25%)	4 (44%)	0.13
*ESR1*	2 (29%)	3 (38%)	1 (11%)	0.44
*MYC*	1 (14%)	2 (25%)	0	0.29

*P < 0.05 is considered significant.

**Table 5:** Number of patients harboring somatic gene alterations according to the site of the sample used for clinical sequencing.

	Primary tumor (n = 13)	Metastatic tumor (n = 11)	*P* value
*TP53*	4 (31%)	3 (27%)	0.85
*PIK3CA*	5 (38%)	2 (18%)	0.28
*AKT1*	2 (15%)	4 (36%)	0.24
*ESR1*	3 (23%)	3 (27%)	0.81
*MYC*	2 (15%)	1 (9%)	0.64

### Overall survival according to genetic alterations

**Figure 1:**
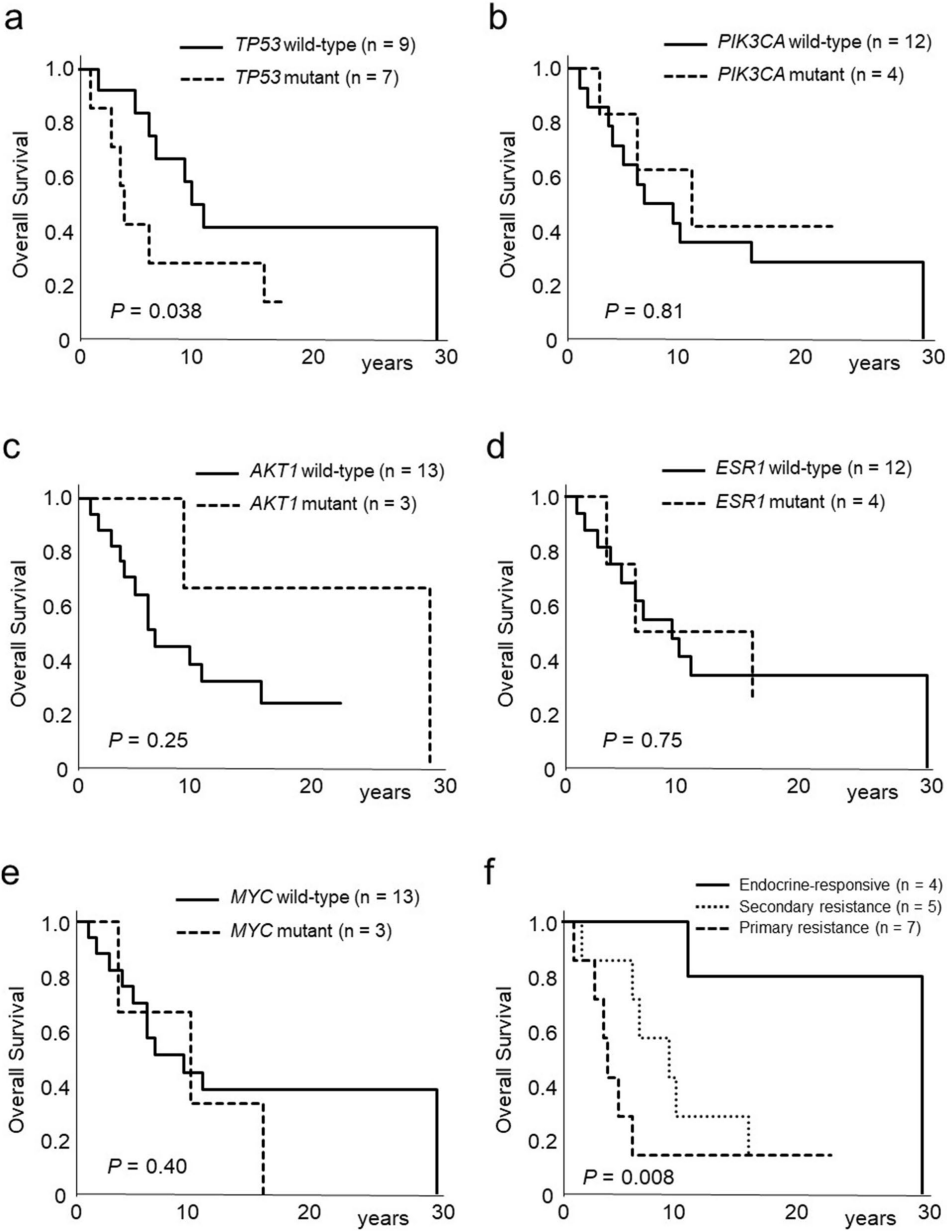
Kaplan–Meier curves of the effect of *TP53* (**a**), *PIK3CA* (**b**), *AKT1* (**c**), *ESR1* (**d**), and *MYC* (**e**) gene alterations for overall survival in recurrent breast cancer patients. (**f**) Kaplan–Meier curves of the effect of endocrine responsiveness for overall survival in recurrent breast cancer patients.

We then determined whether particular sets of genetic alterations correlated with overall survival (time from the diagnosis of primary breast cancer to death) in recurrent breast cancer patients. Kaplan − Meier analysis showed that recurrent breast cancer patients with *TP53*-mutant tumors had significantly worse overall survival compared to those with *TP53*-wild type tumors (*P* = 0.038, Fig. 1a). The presence of alterations in the *PIK3CA*, *AKT1*, *ESR1*, and *MYC* genes was not correlated with overall survival (Fig. 1b–e). Recurrent breast cancer patients with endocrine-responsive disease had significantly longer overall survival compared to those with primary or secondary endocrine-resistant breast cancers (*P* = 0.008, Fig. 1f). Univariate analysis using clinical factors (age, family history, smoking history, initial stage of diagnosis, and endocrine responsiveness) and gene mutation status (*TP53*, *PIK3CA*, *AKT1*, *ESR1*, and *MYC*) demonstrated that endocrine responsiveness was the only significant factor affecting overall survival in recurrent breast cancer patients (*P* = 0.0059).

## Discussion

Since 2019 in Japan, NGS-based panel testing has been covered by insurance for metastatic cancer patients who have not had (or do not respond to) standard treatments. In the present study, we describe our experience of clinical targeted sequencing in ER-positive metastatic breast cancer. The results demonstrated that all of the 24 patients had one or more germline and/or somatic gene alterations. Four (17%) patients with primary or secondary endocrine-resistant breast cancer harbored germline pathogenic variants of *BRCA1*, *BRCA2*, or *PTEN*. *TP53* gene mutations either in the primary or metastatic sites were associated with primary endocrine resistance and poor overall survival.

Large-scale genomic sequencing studies have revealed that ER-positive breast cancer is heterogeneous regarding gene expression, mutation, and copy number variations^4,6^. Somatic gene mutations of *PIK3CA*, *GATA3*, and *MAP3K1* in luminal A subtype, and those of *TP53*, *MDM2*, *PIK3CA*, *PTEN*, and cyclin D1 in luminal B subtype were identified in early breast cancer^4^. On the other hand, gene alterations of *TP53*, *ESR1*, *GATA3*, *KMT2C*, *NCOR1*, *AKT1*, *NF1*, *RIC8A*, and *RB1* were more frequently observed in metastatic breast cancer compared with those in early breast cancer in hormone receptor-positive, HER2-negative disease^7^. This latter report also showed that *TP53*, *RB1*, and *NF1* gene alterations correlated with poor prognosis in hormone receptor-positive, HER2-negative metastatic disease. Furthermore, a study on endocrine-resistant advanced breast cancers revealed that mutations in the mitogen-activated protein kinase (MAPK) pathway, transcriptional regulators of ER (*MYC*, *CTCF*, *FOXA1*, and *TBX3*), and *ESR1* mutations were present in post-endocrine therapy tumors^21^. Identification of the mechanisms of resistance to endocrine therapy in each patient will help inform the use of specific molecular-targeted therapies in order to optimize the therapeutic regimen.

The *TP53* gene is the most frequently mutated gene in all cancers, and is also the most frequent mutation found in breast cancer^4^. A *TP53* gene mutation was present in 12% of luminal A and 32% of luminal B subtypes, although the frequency of *TP53* gene alterations in luminal subtypes is lower than that in basal-like (84%) or HER2-positive (75%) subtypes^4^. Approximately 90% of *TP53* gene alterations encode missense mutant proteins that span ~ 190 different codons located in the DNA-binding domain^22^. Our recent analysis of *TP53* gene alterations in the primary tumors of 56 ER-positive breast cancers showed that all of the 22 mutations existed at different sites^23^. Ellis and colleagues examined somatic gene alterations in pretreatment tumors from patients who were treated with neoadjuvant aromatase inhibitor therapy^24^. They reported that mutations in the TP53 signaling pathway including *TP53*, *ATR*, *APAF1*, or *THBS1* were present in 38% of the aromatase inhibitor-resistant tumors (11 of 29 tumors). We previously reported that p53 protein accumulation in the nucleus, a surrogate marker for *TP53* missense mutation, was a prognostic marker for disease-free survival in patients treated with aromatase inhibitors as adjuvant endocrine therapy^25^. Patients treated with adjuvant endocrine therapy who had *TP53* wild-type tumors also showed longer 5-year overall survival^26^. Furthermore, *TP53* mutations in the primary or metastatic tumors were correlated with poor prognosis in ER-positive metastatic breast cancer^27,28^. Similarly, we showed in the present study that *TP53* mutations either in the primary or metastatic sites affected primary endocrine resistance and poor outcome in ER-positive metastatic breast cancer.

The activation of PI3K/AKT/mTOR signaling pathway is involved in endocrine resistance in ER-positive breast cancer^12,13^. Mutations of the *PIK3CA* gene are frequently observed in luminal A (49%) and luminal B (32%) subtypes^4,29^. Approximately 95% of mutations exist in the helical domain (exon 9, commonly E542 and E545) and the kinase domain (exon 20, commonly H1047)^30^.We and others previously reported that *PIK3CA* mutations were a positive prognostic marker in ER-positive, HER2-negative early breast cancer^31–33^. In contrast, activation of the PI3K pathway is suggested to cause endocrine resistance in ER-positive advanced breast cancer, and therefore inhibitors for PI3K, AKT, and mTOR have potential therapeutic utility in patients with this tumor subtype^12,13^. Furthermore, Mosele and colleagues recently reported that patients with *PIK3CA*-mutated metastatic tumors presented with a poor prognosis and resistance to chemotherapy in hormone receptor-positive, HER2-negative metastatic breast cancer^34^. However, the present study indicated that *PIK3CA* mutations were present in seven tumors (29%) either in the primary or metastatic sites, and that *PIK3CA* mutations were not associated with endocrine responsiveness or overall survival.

On the other hand, somatic *RB1* gene alterations were identified in two tumors in the present study. Loss of RB protein is one of the key mechanisms of CDK4/6 inhibitor resistance^35^. Because a CDK4/6 inhibitor combined with endocrine therapy is recommended as first- or second-line treatments in ER-positive, HER2-negative metastatic breast cancer^36^, information regarding the presence of *RB1* gene alterations was essential for selecting an appropriate treatment strategy.

A survey of real-world outcomes of studies that employed targeted sequencing of small panels (48 to 50 genes) in metastatic breast cancer patients revealed that few patients were admissible to clinical trials with molecular-targeted agents^37^. In contrast, clinical targeted sequencing also led to germline secondary findings. The rate of actionable secondary findings was shown to be from 2 to 5%^37–41^. In the present study, four (17%) out of 24 patients harbored germline pathogenic variants including *BRCA1*, *BRCA2*, or *PTEN*. All four patients with germline pathogenic variants had family members with breast and/or ovarian cancers, whereas germline pathogenic variants were not detected in patients with endocrine-responsive disease. Patients in whom standard treatments are not effective, or whose family members had breast cancer, are likely to undergo targeted sequencing in clinical practice. Furthermore, one patient in the present study with no germline *BRCA1* pathogenic variants had a primary tumor with a somatic *BRCA1* gene mutation. De novo somatic *BRCA1/2* mutations in breast cancer are considered to be rare: the prevalence of somatic *BRCA1* gene mutations in primary tumors is only 1.55%, and that of somatic *BRCA2* gene mutations is 1.68%^42^. Although the clinical success of PARP inhibitors for women with germline *BRCA1/2* pathogenic variants is clear, the utility of using PARP inhibition to treat breast cancers with somatic *BRCA1/2* mutations is not known.

The present study has several limitations. First, the size of our cohort was too small for a valid statistical analysis and to provide reliable real-world evidence. Furthermore, the site of samples used for clinical sequencing was either the primary or metastatic tumor, because it is often difficult to obtain samples from metastatic sites in clinical practice. However, we do show details of the characteristics, treatments and gene alterations of each patient (Tables 2 and 3). Because 24 samples were all the cases that we experienced during the study period, it is difficult to set an independent dataset to replicate the original findings. It is necessary to construct a prediction model for endocrine therapy based on the results of gene mutations^43^. Using the deep learning method will help to predict endocrine resistance and the efficacy of targeted therapies for precision medicine in ER-positive breast cancer^44^.

Second, our clinical targeted sequencing with 160 cancer-related genes is not breast cancer-specific. Thus, some genes essential for breast cancer progression, especially germline variants, might not be covered in this system. Third, the estimation of alterations of each gene has been improving over time. Consequently, we reanalyzed the sequencing results with reference to the newest versions (the Ensembl Variant Effect Predictor (version 95) for somatic variants and ClinVar for germline variants).

In this study, we report our clinical experience of targeted sequencing in ER-positive, HER2-negative metastatic breast cancer. Germline and somatic gene alterations as well as patients’ prognosis were retrospectively analyzed, and we determined whether mutation patterns were correlated with a particular response to endocrine therapy. Our study demonstrated that 17% of patients with primary or secondary endocrine-resistant breast cancer harbored germline pathogenic variants of *BRCA1*, *BRCA2*, or *PTEN*. *TP53* gene mutations were affected by primary endocrine resistance and poor overall survival. Our 160-gene cancer panel will be useful to identify clinically actionable gene alterations in patients with endocrine-responsive and endocrine-resistant breast cancer in clinical practice. Accumulation of real-world data on clinical targeted sequencing from individual patients will help to select appropriate therapies in endocrine-resistant breast cancer.

## Methods

### Patients and clinical targeted sequencing

Between April 2016 and March 2018, 24 patients with ER-positive, HER2-negative metastatic breast cancer underwent comprehensive genomic testing with either our original in-house CLHURC system^16–20^ or with OncoPrime (EA Genomics, Morrisville, NC, USA). In the CLHURC system, genomic DNA was extracted both from tumor tissue and peripheral blood mononuclear cells obtained from cancer patients, who provided consent to undergo comprehensive genomic testing. After checking the quality of the DNA based on the DNA integrity number (DIN) score calculated using the Agilent 2000 TapeStation (Agilent Technologies, Waldbronn, Germany), we performed a targeted amplicon exome sequencing for 160 cancer-related genes using the Illumina MiSeq sequencing platform (Illumina, San Diego, CA, USA). The list of 160 cancer-related genes included in the CLHURC comprehensive cancer panel was shown in the previous study^19,20^. The minimum amount of DNA was 50 ng and the minimum quality of DNA had a DIN score over 3.1. This study was approved by the Ethics Committee of the Hokkaido University Graduate School of Medicine (No. 18–049), and conformed to the guidelines of the 1996 Declaration of Helsinki. Informed consent was obtained from each patient.

### Bioinformatics analysis

Data from the MiSeq runs were processed initially using the MiSeq platform-specific software MiSeq Reporter (Illumina) to generate sequence reads as a FASTQ file. The generated FASTQ files were then processed with Trim-galore (https://www.bioinformatics.babraham.ac.uk/projects/trim_galore/) for trimming adapter sequences and filtering and remove poor signal-profile reads. Burrows-Wheeler Aligner (BWA)^45^ was used to align the paired-end reads to the hg19 human reference genome to generate BAM format files. BAM files were coordinately sorted and indexed with SAMtools (v1.6)^46^. Indels in sequence alignment files were left-aligned, and local realignment around Indels was done with the Genome Analysis ToolKit (GATK 3.4–46) RealignerTargetCreator and IndelRealigner^47^. Base quality score recalibration was performed with GATK tools. For checking sequence run quality, Illumina InterOp library (https://illumina.github.io/interop/index.html) was used. FASTQ files were quality controlled using FastQC (https://www.bioinformatics.babraham.ac.uk/projects/fastqc/) (v0.11.6), and BAM files were evaluated those qualities with GATK DepthOfCoverage and Qualimap 2 (v2.2.2)^48^. VarScan 2^49^ was used to identify single nucleotide variants and small insertions/deletions. If the sample had the normal control data, tumor-normal pair analysis was conducted for calling variants. Copy number variants were identified using CNVkit^50^ with matched normal. If without normal, Panel of Normal dataset was used as the reference. For calling somatic variant, possible germline single nucleotide polymorphisms (SNPs) were excluded using Single Nucleotide Polymorphism Database (dbSNP) (https://www.ncbi.nlm.nih.gov/snp/), the gnomAD database (https://gnomad.broadinstitute.org/) and the ToMMo 4.7KJPN database (https://jmorp.megabank.tohoku.ac.jp/ijgvd/). Variant filtering was conducted using bcftools^51^ with alternate allele count, variant allele frequency and read depth and normal pool dataset created before this study, followed by manual review with Integrative Genomics Viewer (Version2.3.57)^52^ and BAF-LogR profiles. We have used the Ensembl Variant Effect Predictor (version 95)^53^ for genomic and functional annotation of the variants.

Germline variants were classified according to the American College of Medical Genetics and Genomics (ACMG) guidelines^38^, and retained as pathogenic with conflicting interpretation in ClinVar (https://www.ncbi.nlm.nih.gov/clinvar/)^54^. The results of germline secondary findings were disclosed to patients only who agreed to disclose germline information.

### Endocrine responsiveness in metastatic breast cancer

We defined endocrine responsiveness according to ABC 4^14^ as follows: Primary endocrine resistance was defined as relapse while on the first 2 years of adjuvant endocrine therapy, or progressive disease (PD) within first 6 months of first-line endocrine therapy for advanced breast cancer, while on endocrine therapy. Secondary endocrine resistance was defined as relapse while on adjuvant endocrine therapy but after the first 2 years, or relapse within 12 months of completing adjuvant endocrine therapy, or PD within 24 months of first-line endocrine therapy for advanced breast cancer. Endocrine-responsive breast cancer was defined as relapse after 12 months of completing adjuvant endocrine therapy, or PD ≥ 24 months of first-line endocrine therapy for advanced breast cancer.

### Statistical analysis

The significance of differences among three groups was evaluated using the chi-squared test. *P* values < 0.05 were considered significant. Estimation of overall survival (time from the diagnosis of primary breast cancer to death) was performed using the Kaplan–Meier method, and differences between survival curves were assessed using the log-rank test. The Cox proportional hazards model was used for prognostic analysis. Statistical analysis was performed using Excel software (Microsoft corp., Albuquerque, MX, USA).
